# In silico epitope mapping and experimental evaluation of the Merozoite Adhesive Erythrocytic Binding Protein (MAEBL) as a malaria vaccine candidate

**DOI:** 10.1186/s12936-017-2144-x

**Published:** 2018-01-10

**Authors:** Pedro Cravo, Renato B. Machado, Juliana A. Leite, Taizy Leda, Rossarin Suwanarusk, Najara Bittencourt, Letusa Albrecht, Carla Judice, Stefanie C. P. Lopes, Marcus V. G. Lacerda, Marcelo U. Ferreira, Irene S. Soares, Yun Shan Goh, Daniel Y. Bargieri, François Nosten, Bruce Russell, Laurent Rénia, Fabio T. M. Costa

**Affiliations:** 1 Global Health and Tropical Medicine Centre (GHTM), Instituto de Higiene e Medicina Tropical (IHMT), Universidade Nova de Lisboa, Rua da Junqueira, nº 100, 1349-008 Lisbon, Portugal; 2 GenoBio, Instituto de Patologia Tropical e Saúde Pública, Universidade Federal de Goiás, Goiânia, GO Brazil; 3 PPG-SOMA, Centro Universitário de Anápolis, Anápolis, GO Brazil; 4 Laboratory of Tropical Diseases-Prof. Dr. Luiz Jacintho da Silva, Department of Genetics, Evolution, Microbiology and Immunology, University of Campinas-UNICAMP, Campinas, SP Brazil; 5 Singapore Immunology Network, Agency for Science, Technology and Research (A*STAR), Singapore, Singapore; 6 Instituto Carlos Chagas, Fundação Oswaldo Cruz-FIOCRUZ, Curitiba, PR Brazil; 7 Instituto Leônidas e Maria Deane, Fundação Oswaldo Cruz-FIOCRUZ, Manaus, AM Brazil; 8 Fundação de Medicina Tropical-Dr. Heitor Vieira Dourado, Gerência de Malária, Manaus, AM Brazil; 9 Department of Parasitology, University of São Paulo-USP, São Paulo, SP Brazil; 10 Department of Clinical and Toxicological Analyses, Pharmaceutical Sciences, University of São Paulo-USP, São Paulo, SP Brazil; 11 Department of Microbiology, National University of Singapore-NUS, Singapore, Singapore; 12 Shoklo Malaria Research Unit, Mahidol-Oxford Tropical Medicine Research Unit, Faculty of Tropical Medicine, Mahidol University, Mae Sot, Thailand; 13 Centre for Tropical Medicine, Nuffield Department of Medicine, University of Oxford, Oxford, UK

## Abstract

**Background:**

Technical limitations for culturing the human malaria parasite *Plasmodium vivax* have impaired the discovery of vaccine candidates, challenging the malaria eradication agenda. The immunogenicity of the M2 domain of the Merozoite Adhesive Erythrocytic Binding Protein (MAEBL) antigen cloned from the *Plasmodium yoelii* murine parasite, has been previously demonstrated.

**Results:**

Detailed epitope mapping of MAEBL through immunoinformatics identified several MHCI, MHCII and B cell epitopes throughout the peptide, with several of these lying in the M2 domain and being conserved between *P. vivax*, *P. yoelii* and *Plasmodium falciparum*, hinting that the M2-MAEBL is pan-reactive. This hypothesis was tested through functional assays, showing that *P. yoelii* M2-MAEBL antisera are able to recognize and inhibit erythrocyte invasion from both *P. falciparum* and *P. vivax* parasites isolated from Thai patients, in ex vivo assays. Moreover, the sequence of the M2-MAEBL is shown to be highly conserved between *P. vivax* isolates from the Amazon and Thailand, indicating that the MAEBL antigen may constitute a vaccine candidate outwitting strain-specific immunity.

**Conclusions:**

The MAEBL antigen is promising candidate towards the development of a malaria vaccine.

**Electronic supplementary material:**

The online version of this article (10.1186/s12936-017-2144-x) contains supplementary material, which is available to authorized users.

## Background

Malaria is one of the most nefarious infectious diseases of humans and continues to have a devastating global impact upon health and well-being, mainly among children under the age of five and pregnant women. Annually, about 200 million cases are reported and almost 600,000 deaths occur [1]. Despite the recent relative progress towards a *Plasmodium falciparum* vaccine, the development of a vaccine to protect individuals from *Plasmodium vivax* is still incipient, jeopardizing the Malaria Vaccine Roadmap [2], and consequently the whole malaria eradication agenda. Currently, the difficulty to grow *P. vivax* in vitro for long periods [3] challenges ongoing strategies for identification of novel vaccine candidates against this parasite. Considering these roadblocks, an approach to develop vaccines against *P. vivax* might rely on the use of malaria antigens conserved amongst species.

Recently, the immunogenicity of M2 MAEBL (Merozoite Adhesive Erythrocytic Binding Protein) domain of *Plasmodium yoelii* has been demonstrated. This vaccine candidate conferred 90% protection in immunized mice after lethal challenge and corresponding antisera inhibited significantly erythrocyte invasion by *P. yoelii* [4]. MAEBL is a membrane protein that belongs to the erythrocyte binding protein (*ebl*) family [5–8] and exhibits homologous regions to the DBL-EBP (Duffy binding like–Erythrocyte binding protein) and to the apical membrane antigen 1 (AMA1) [7–9]. MAEBL is a conserved antigen expressed in blood-stage late forms, in the salivary gland sporozoite and also during the late liver stage [10, 11].

Immunoinformatics, in which computational approaches select the most appropriate vaccine candidate and algorithms predict T- and B-cell immune epitopes [12], is an alternative to overcome limitations imposed by the lack of knowledge in terms of *P. vivax* biology.

Here, an immunoinformatics strategy was used, that identified the MAEBL antigen as a promising interspecies and interstrain malaria vaccine candidate and the pan-reactivity of the *P. yoelii* M2-MAEBL antisera against *P. falciparum* and *P. vivax* was investigated.

## Methods

### B-cell and T-cell epitope prediction

#### Fine epitope mapping through bioinformatics

The predicted entire MAEBL protein sequence of the rodent malaria parasite *P. yoelii*, available at PlasmoDB (PYYM_0902200.1) was initially used to predict C57BL/6 J mouse MHC epitopes for H-2Kb, H-2Db (MHC class I) and IA-b alleles (MHC class II). Predictions for linear B lymphocyte epitopes were also ran. Detailed procedures were as follows.

#### MHC class I and II epitope mapping

Rankpep software [13–16] set to a binding threshold of 3% and proteasome cleavage filter “ON” was used for initial MHC class I epitope screening. Subsequently, the MAEBL protein was re-screened using IEDB [17, 18], NetMHCpan [19, 20], Bimas [21, 22], MAPPP [23, 24] and PropredI [25]. All epitopes that were identified using all softwares were thus selected as those presenting the highest confidence.

Rankpep was also used to predicted MHC class II epitopes, with a binding threshold set to 3% and proteasome cleavage filter “OFF”. Resulting predicted epitopes with a score above 9.52 was selected for further analyses using IEDB [17, 18] and NetMHCII [26–28]. Epitopes displaying the best scores generated between all screens were included in the final list.

#### B-cell epitope mapping

The BCPRED resource [29, 30] was employed to identify B-cell epitopes with a size of 20 aminoacids, set to a 75% specificity. After identifying the best predicted epitopes based on score, each of these epitopes was screened for predicted antigenicity using the VaxiJen v.2 software [31], under a 0.5 threshold and the “parasite model” filter on, according to previously published recommendations [32, 33]. Subsequently, all epitopes presenting scores above 0.5 were included in the final list. Lastly, the potential conservation within each of these epitopes between *P. yoelii* and *P. falciparum,* and between *P. yoelii* and *P. vivax* was investigated, using the BLAST tool at PlasmoDB, and epitopes were considered homologous between P. *yoelii* and any of the other two human parasites when amino acid identity was higher than 50%.

### Immunization regimen

C57BL/6 mice with 5–7 weeks-old were injected subcutaneously four times at 3 weeks intervals with 5 μg of rPyM2-MAEBL emulsified in 1:1 (vol/vol) complete Freund’s adjuvant (CFA) for the first dose or incomplete Freund’s adjuvant (IFA) in the subsequent doses [4].

The prime-boost (PB) group received the first dose of 100 μg pIgSPM2 intramuscular, followed by three doses of 5 μg of rPyM2-MAEBL in IFA. As control groups, animals were injected with 1:1 (vol/vol) adjuvant (CFA/IFA), pIgSPM2 or only pIgSP vector. Sera from immunized mice were collected immediately before each dose and 3 weeks after the last dose. There was no significantly difference in protection between the rM2-MAEBL and the prime-boost regimen. All experiments and procedures were performed in accordance with relevant guidelines and regulations of the Ethical Committee for Animal Research of the University of Campinas and were approved under Protocol No. 1437-1.

### Slide preparation and immunofluorescence assays (IFA)

Clinical isolates of *P. vivax* and *P. falciparum* infected blood from malaria patients were collected at Shoklo Malaria Research Unit (Thailand) with written informed consent. The thin smears used for the IFA were prepared from ex vivo matured schizonts concentrated by 45% Percoll for *P. vivax* and 70% Percoll for *P. falciparum* [3, 34] that were diluted 1:4 with uninfected RBCs. Immunofluorescence assays were performed after fixing the blood smears with ice-cold acetone for 20 min and air-dried. Well diameters were established with the aid of a Dako-Pen (Dako), and blocking was performed by 30-min incubation at 37 °C with PBS containing 3% BSA (USB). C57BL/6 mice with 5–7 weeks-old were immunized as described elsewhere [4] and pooled sera from the different immunization groups were diluted 1:50 in PBS supplemented with 3% BSA and applied to the slides for 1 h at 37 °C. Slides were washed 3X in PBS and incubated with Alexa-568 goat anti-mouse IgG (Invitrogen) for 1 h at 37 °C in the dark, then washed 3X in PBS and incubated with DAPI (4′,6-diamidino-2-phenylindole, dihydrochloride-(Invitrogen) diluted in ultrapure (Millipore) water for 10 min at room temperature. After another round of washing, Fluorosave (Caliobiochem) was added, and slides were sealed with coverslips. Parasites were visualized with the aid of a Nikon TS100 epifluorescence microscope. All samples above were collected in accordance with relevant ethical guidelines and regulations of the University of Oxford, Centre for Clinical Vaccinology and Tropical Medicine and the Ethics committee of Faculty of Tropical Medicine, Mahidol University, under the approved protocols OXTREC 027-025 and MUTM 2008-215 from.

### Plasmodium-specific antibody binding assay

#### Transfection

Transfection of HEK293 cells to obtain cells expressing PvMAEBL, PfMAEBL, PfSEA or PVX_113775 on the cell surface was as previously described [35]. Nucleotide sequences encoding for PvMAEBL (PVX_092975: amino acid 540–1007), PVX_113775 (amino acid 1–358), PfMAEBL (PF3D7_1147800: amino acid 958–1249) and PfSEA (PF3D7_1021800: amino acid 810–1083) were amplified via PCR, using either *P. vivax* UMS203 or *P. falciparum* 3D7 RNA as template, and cloned into the pDisplay vector (Invitrogen). The resultant plasmids were then transfected into HEK293 cells using lipofectamine 2000 (Invitrogen) for surface expression of the antigens.

#### Antibody binding assay

The antibody binding assay was as previously described [35]. Briefly, transfected cells, expressing the antigens on the cell surface, were first incubated with the serum (diluted 1:100 in FACS blocking buffer (10% FBS in PBS) on shaking. The cells were then incubated with a double stain, consisting of Alexa Fluor 488-coupled secondary antibodies (Invitrogen; diluted 1:500) and propidium iodide (PI; diluted 1:2500) on shaking. Cells were read on Accuri C6 (BD Biosciences) and analysed using FlowJo (Tree Star).

### Ex-vivo functional invasion assays

#### *Plasmodium falciparum* isolates

The preparation of schizonts concentrate after 70% Percoll was mixed with target cells (RBCs/uninfected erythrocytes) in the ratio 1:12. The solution was diluted with 2% haematocrit using McCoy 5A 10% human AB serum (inactivated) and cultivated in microplates in a volume of 200 µl, 5% O2 at 37.5° C for 12 h. The inhibitory potential of anti-PyM2-MAEBL antibodies were tested by adding the fourth pool of sera from animals immunized with a dose of the rPyM2-MAEBL protein alone or in heterologous prime-boost system at the 1:50 dilution to the final mixture invasion assay. It was used as a control serum pool of the 4th dose animals of ACF/AIF group. As positive control, it was used 100 μM of E64 (Sigma), a protease inhibitor, to ensure no disruption of schizonts. At the end of the test were made blood smear slides of each well stained with Giemsa (Sigma). The number of rings/trophozoites to 1000 cells was determined by microscopic analysis.

#### *Plasmodium vivax* isolates

20 ml of cord blood were collected in heparin tube immediately after the newborn delivery. Samples were collected after informed consent was obtained from each subject and in accordance with relevant guidelines and regulations of the ethics committee of the Fundação de Medicina Tropical—Dr. Heitor Vieira Dourado (protocol CAAE-0044.0.114.000-11).

The blood group was determined by using a standard ABO Kit (EBRAN). After plasma removal, the cells were washed in McCoy 5A medium (Sigma) and haematocrit adjusted to 50% using McCoy 5A. Leukocytes and platelets were depleted by two passages in CF11 filtration column (Whatman). Red cells from umbilical cord were again adjusted to 50% haematocrit and carefully overlaid on tubes containing 70% Percoll (GE Healthcare). According to Russell et al. [3], the preparation of schizonts concentrate after 45% Percoll enrichment was mixed with target cells (cord blood) in the proportion 1: 6. The solution was diluted to 2% haematocrit using McCoy 5A (Sigma) 20% human AB serum (inactivated) and cultivated in microplates in a volume of 200 µl 5% O2 at 37.5 °C for 24–30 h depending on the parasite maturation. The inhibitory potential of anti-PyM2-MAEBL antibodies were tested by adding the fourth dose pool sera from animals immunized with rPyM2-MAEBL protein alone or in heterologous prime-boost system at the 1:50 dilution to the final mixture invasion assay. It was used as a control sera pool of the 4th dose animals of ACF/AIF group. As a positive control of inhibition was used polyclonal anti-*P. vivax* Duffy Binding Protein (PvDBP) IgG [36]. At the end of the test blood smear slides of each well were performed and stained with Giemsa (Sigma). The number of rings/trophozoites from 1000 cells was determined by microscopic analysis.

### Amplification and *maebl* sequencing from Amazonian isolates

**Fig. 1:**
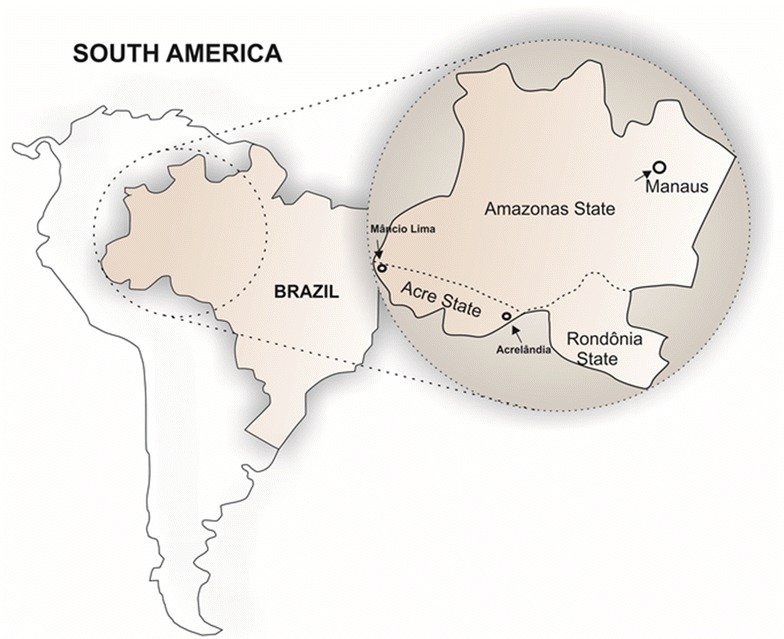
Geographic areas in the Amazon where *P. vivax* samples were collected. Manaus, Mâncio Lima and Acrelândia indicated by arrows. The map was generated by the authors using CorelDRAW graphics suite X7 software

A total amount of nine blood samples were collected (during the period of 2012–2014) from different areas in the Amazon: the cities of Manaus in the Amazonas state and Mâncio Lima and Acrelândia, both in state of Acre (Fig. 1). The identification of *P. vivax* species was performed by nested-PCR as previously described [37]. For Pv-MAEBL amplification, oligonucleotides were designed based on the Pv-MAEBL Sal-1 strain (PVX_092975) sequence from PlasmoDB. Three DNA fragments were PCR-amplified to obtain the sequence of M2-MAEBL domain. Oligonucleotide sequences used in this study are displayed in Additional file 1. Reactions to amplify fragments one and three were performed with Platinum *Taq* (Invitrogen). Amplification condition was as follows: 1 cycle of 5 min at 95 °C and 35 cycles of 30 s at 95 °C, 45 s at 58 °C, 1 min at 72 °C and a final cycle of 5 min at 72 °C. The fragment two was amplified using Phusion DNA Polymerases (Thermo Fisher Scientific). The PCR reaction was submitted to 1 cycle of 5 min at 98 °C and 35 cycles of 15 s at 98 °C, 30 s at 59 °C, 1 min at 72 °C and a final cycle of 10 min at 72 °C. The purified PCR product was sequenced using 3730 × l DNA Analyzer (Applied Biosystems). All generated sequences were subjected to similarity search analysis by BLAST.

### Alignment of the M2 *maebl* domain sequences of *P. vivax*

The MAEBL M2 domain of the nine isolates from Brazilian Amazon (GenBank accession nos. KX061004 to KX061012) were compared to previously described sequences deposited in PlasmoDB: Thailand_VKBT-101, Thailand_VKBT-100, Thailand_VKTS-39 and the reference sequence Pv-MAEBL Sal-1 strain (PVX_092975), using the Clustal Multialin Interface Page [38].

## Results

### Detailed in silico epitope mapping of the *P. yoelii* Merozoite Adhesive Erythrocytic Binding Protein (MAEBL)

#### MHC class I and II epitope mapping

In silico epitope mapping identified two predicted epitopes for MHC class I, for each of the H-2Kb and H-2Db alleles (Additional file 2). Epitope QNYYSFTNL for the H-2Kb allele presented the highest score between all programs used, whereas the NQNINLVKL epitope for allele H-2Db was among the top ten with the highest scores. Initial epitope screening for MHC class II using Rankpep resulted in the identification of 19 epitopes. However, after further analysis with IEDB and NetMHCII, only two epitopes were common between all softwares (Additional file 2).

#### B-cell epitope mapping

**Fig. 2:**
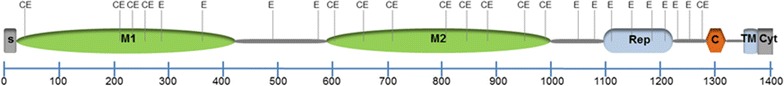
Schematic representation of the *P. yoelii* MAEBL antigen with locations of domains and in silico mapped B-cell epitopes. *S* Signal peptide, *Rep* Repetitive Domain; *C-* Cysteine-rich Domain, *TM* Transmembrane Domain, *Cyt* Cytoplasmic Domain, *E* B-cell epitope, *CE* conserved B-cell epitope

Using BCPRED, 35 predicted epitopes with a score above 0.8 were identified. Each of these was further analysed using Vaxijen software in order to confirm antigenicity, resulting in a total of 25 epitopes with a score higher than 0.5 (Additional file 3). BLAST searches with these epitopes against human malaria parasite sequences revealed that 12 epitopes present homology to both *P. falciparum* and *P. vivax* sequences. Although epitopes were largely scattered evenly throughout the peptide (Fig. 2), interestingly, 8 out of these 12 conserved epitopes fall within the M2 domain of MAEBL (Additional file 3, Fig. 2).

### Immunofluorescence and invasion inhibition assays with *P. falciparum* and *P. vivax* isolates

**Fig. 3:**
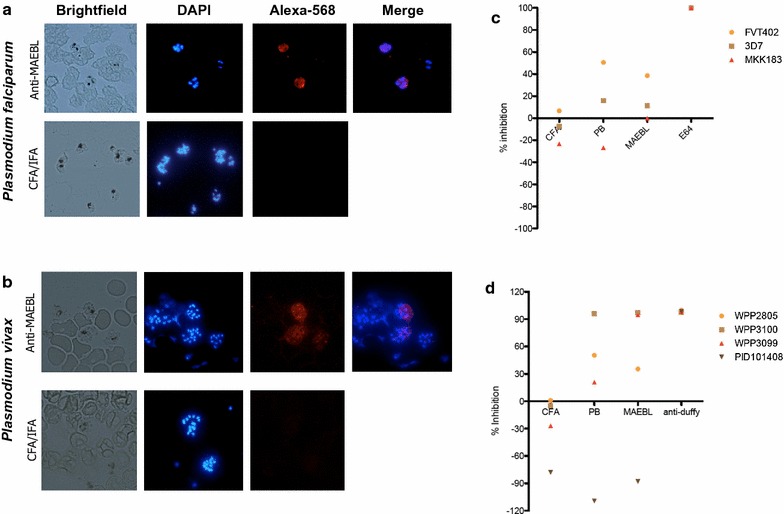
*Plasmodium yoelii* MAEBL antisera pan-reactivity against human malaria parasites. **a** rPyM2MAEBL antibodies recognize *Plasmodium* falciparum (3D7 strain) schizonts. **b** rPyM2MAEBL antibodies also recognize *Plasmodium vivax* schizonts. Sera pool from the 4th dose of animals immunized with rPyM2MAEBL, prime-boost regimen or of mice injected with adjuvant only (CFA/IFA) were used at a dilution of 1:50 in indirect immunofluorescence assays against *P. falciparum* or *P. vivax* schizonts. Sera from mice immunized with rPyM2-MAEBL or heterologous prime-boost system inhibit invasion by **c** *P. falciparum* to normocytes and **d** *P. vivax* to reticulocytes. Schizonts and target cells were cultured in vitro in the presence of sera pool from the fourth dose of immunized mice diluted 1:50. As a positive control for inhibition in *P. vivax* assays anti-Duffy polyclonal sera was used in 1:50 dilution. In *P. falciparum*, E64 protease inhibitor (Sigma) was used as positive control of inhibition. For both species sera from mice injected with CFA/IFA were used as a negative control. The parasitaemia was determined by counting at least 1000 red blood cells. The results are expressed as percentage inhibition compared to the control without sera

**Fig. 4:**
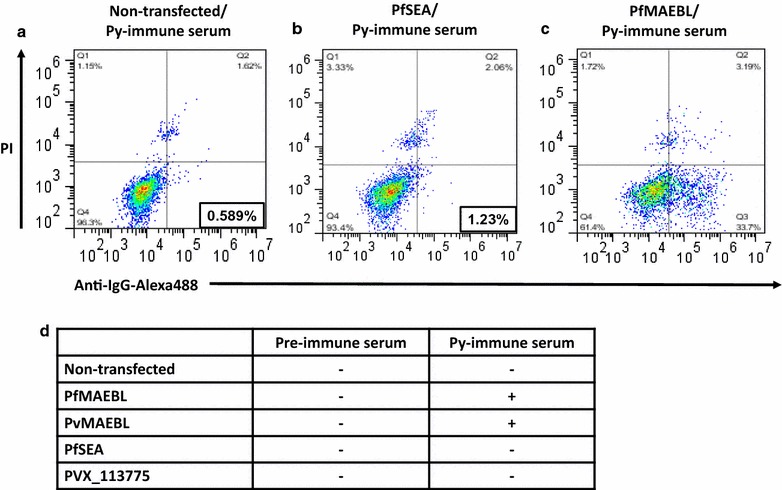
Serum response against PfMAEBL and PvMAEBL. Pre-immune and PyMAEBL-immune mouse serum were probed against non-transfected HEK293 cells (negative control), HEK293 cells transfected for cell surface expression of PfSEA (negative control for Pf), PVX_113775 (negative control for Pv), PfMAEBL or PvMAEBL and analysed by FACS. FL1 signal indicates specific binding (Alexa Flour 488 staining) while FL2 signal indicates dead cells. Representative plots for positive and negative serum response shown (**a**–**c**). As negative control, background IgG response (in black box) was observed **a** with PyMAEBL-immune serum against non-transfected live cells and **b** with PyMAEBL-immune serum against live cells expressing PfSEA, an antigen with no homology to MAEBL. **c** Positive IgG binding was observed with live cells expressing PfMAEBL (in black box). **d** Serum response for all constructs tested for pre-immune and PyMAEBL-immune serum

Bearing in mind the conserved feature of MAEBL amongst *Plasmodium* species, investigated the ability of *P. yoelii* M2-MAEBL antisera pool was investigated as previous described [39], to recognize blood schizonts of *P. falciparum* (3D7) and *P. vivax* harvested from infected patients. Their reactivity was analysed by indirect immunofluorescence assay (IFA). As shown in Figs. 3a, b, *P. yoelii* antisera raised against M2-MAEBL recombinant protein cross-react with both human parasites. Although the pattern observed at the IFA (Fig. 3a, b) is different from the one previously observed at the Leite et al. [4]. As a control, sera from mice injected with complete/incomplete Freund’s adjuvant (CFA/IFA) were used. To further validate the pan reactivity of the anti-sera raised against the M2 region of the PyMAEBL protein the sera against HEK cells expressing on their surface PvMAEBL or PfMAEBL fragments or unrelated antigens was tested. Only HEK cells expressing the PvMAEBL and the PfMAEBL showed reactivity (Fig. 4). Next, to verify if M2-MAEBL antisera cross-reactivity could interfere on the parasite invasion process to erythrocytes, functional ex vivo invasion assays using two *P. falciparum* and four *P. vivax* isolates were performed. The MAEBL antisera inhibited up to 40–60% invasion of the FVT402 *P. falciparum* isolate; however, no inhibition was observed for the MKK183 isolate (Fig. 3c). Nevertheless, Prime Boost (PB) (described in “Methods” section) or MAEBL antisera inhibited at higher levels the FVT402 isolate than the 3D7 *P. falciparum* culture. As expected, no newly invaded erythrocytes were observed when the protease inhibitor E64 was added to the assays. Functional invasion analyses using different *P. vivax* isolates (Fig. 3d) demonstrated that *P. yoelii* M2-MAEBL antisera (PB and MAEBL) inhibited erythrocyte invasion by most of the isolates tested. Inhibition levels (up to 90%) observed were comparable to anti-Duffy antibodies, which block the DARC receptor essential for *P. vivax* invasion, however no statistical difference was observed. In general, PB antisera inhibited invasion at higher levels than MAEBL, probably due to the difference between immunization regimens. No inhibition was observed in the presence of sera from mice immunized with only CFA/IFA.

### Alignment of the M2 *maebl* domain sequences

The M2 *maebl* domain of *P. vivax* of nine patients from three different areas in the Amazon was sequenced (Additional file 4). The isolates were compared to three Thai isolates as well as the reference strain Sal-1 sequences previously deposited in PlasmoDB. A high degree of similarity was observed among all sequences, with only two SNPs differentiating the Brazilian and Sal-1 sequences from Thai parasites (Additional file 4). These findings may largely explain the similar inhibitory levels observed in functional assays amongst different isolates and reinforce the validity of using the *P. vivax* Sal-1 strain genome available at PlasmoDB for immunoinformatics analysis.

## Discussion

Functional assays from previous work have suggested that MAEBL is a suitable malaria vaccine candidate [4]. For this reason, MAEBL was studied in further detail here, through a conjugation of detailed epitope mapping in silico and further experimental evaluation. Epitope mapping identified four MHC classes I and II putative epitopes within the *P. yoelii* MAEBL antigen, which resulted from a consensus between different prediction programs. It has been shown that MAEBL is expressed not only in blood stages, but also in midgut and salivary gland sporozoites [11, 40, 41] suggesting that it may be important for the establishment of a successful pre-erythrocytic infection of the parasite. As such, in light of the discovery of a malaria vaccine, selecting antigens that present MHC class I and II epitopes should be prioritized given the mounting evidence that antigen-specific CD8+ and CD4+ immune responses contribute for protection from sporozoite challenge both in animal models and human malaria [42]. In addition, it was determined that the MAEBL antigen contains at least 25 predicted B-cell epitopes that are likely to elicit antibody-dependent immune responses, which have been widely shown to be important for protection against blood-stage antigens [42]. Although these epitopes were scattered throughout different domains of MAEBL, more than half of them (fourteen) are located within the M1 and M2 ligand domains, which have been confirmed to bind to mouse erythrocytes [9], suggesting their role in cell invasion. Moreover, all but one of these fourteen epitopes lying in the M1 and M2 domains are conserved between *P. yoelii*, *P. vivax* and *P. falciparum*. It has been shown that a particular epitope (YVSSFIRPDYETKCPPRYPL) present in the *P. falciparum* MAEBL M2 domain is highly immunogenic and capable of binding human HLA with high avidity [43]. Interestingly, the present bioinformatics predictions identified an epitope with a very similar sequence (SSFIRPDYETKCPPRYPL) that is conserved between *P. yoelii*, *P. falciparum* and *P. vivax*. Since the M2 peptide was used here in the immunization experiments, the extant conservation of this and the other epitopes may largely explain the pan-reactivity observed and further suggests that this peptide may be a promising candidate for a malaria vaccine targeting more than one parasite species. In fact, previous experimental MAEBL epitope mapping revealed that anti-MAEBL IgM and IgG antibodies displayed strong responses against fragments located in the M2 region [4].

*Plasmodium* merozoites may use multiple pathways to invade red blood cells. The latter point is supported by data from previous experiments in which MAEBL knockout *Plasmodium berghei* strain ANKA parasites were still able to invade red blood cells in vivo [11]. Although erythrocyte invasion by *Plasmodium* merozoites seems to be largely host-specific, different *Plasmodium* species keep several structurally and functionally homologous adhesins that play important roles during invasion, which enable them to, in some cases, invade red cells of heterologous host species [44]. It was shown that proteins from *P. falciparum* and *P. yoelii* bound to heterologous host red cell receptors, likely through their conserved motifs; suggesting homologies between *Plasmodium* ligands [44]. Bioinformatics analyses show high similarities between the functional and genomic sequences across *Plasmodium* species [45, 46]. Also, studies suggest preservation of erythrocyte binding function of phylogenetically distant *Plasmodium* species [45]. These observations indicate that interspecies conserved epitopes that map within conserved functional motifs could be promising candidates for the development of effective vaccines.

MAEBL is an antigen expressed during the blood and liver stages and is essential for sporozoite invasion of mosquito salivary glands [11]. MAEBL is also implicated in the invasion of merozoites into new erythrocytes, though not essential at this stage. The ability of anti-MAEBL antibodies to inhibit the invasion of merozoites of *P. falciparum* was demonstrated here. Considering that MAEBL seems to be a highly conserved antigen and expressed at different parasite stages, determination of its potential as a vaccine is paramount. Indeed, it has recently been shown that M2 MAEBL immunization confers 90% mice survival against lethal challenge with *P. yoelii* parasites [4].

Furthermore, the pan-reactivity of the anti-sera raised against rPyM2-MAEBL was revealed, which is based on the M2 domain of the *P. yoelii* MAEBL antigen. In addition, the fact that HEK cells expressing the PvMAEBL and the PfMAEBL showed reactivity presents a counter proof of the pan-reactivity of the anti-sera raised against the PyMAEBL M2 region and thus validated the ex vivo assays. An IFA pattern different from the article published by Leite et al. Was detected [4]. Here, a cytosolic staining was observed and previously an apical staining was described. This difference could be justified by the fact that the anti-serum could recognize different *Plasmodium* proteins. These antibodies were able to recognize and inhibit at high levels the invasion of *P. falciparum* and *P. vivax* (mainly) collected from infected patients in Thailand, suggesting that antibodies against one *Plasmodium* species recognize several antigens from other species [34, 47, 48] and the presence of homologous molecules with interspecies conserved epitopes.

## Conclusions

Combined immunoinformatics and experimental approach strategies confirmed the potential of the MAEBL antigen as a malaria vaccine candidate. Whilst the MAEBL may be unlikely to confer sterile immunity if used as the sole component of a vaccine, it may be combined with other antigens towards the synthesis of a fully effective chimeric subunit vaccine. Moreover, the *P. yoelii* mouse model can be used to test human malaria parasite antigens for their immunogenicity and efficacy as vaccine candidates.

## Additional files

**Additional file 1.** MAEBL Oligonucleotide position and sequences. (# The nucleotide positions are based on *Plasmodium vivax* strain Sal-1 MAEBL sequence (PVX_092975) available at PlasmoDB).

**Additional file 2.** Predicted MHC class I and II epitopes within the *Plasmodium yoelii* MAEBL antigen generated from a consensus between different epitope prediction programs.

**Additional file 3.** Predicted *Plasmodium yoelii* B-cell epitopes within the putative MAEBL antigen. (Epitopes were generated using BCPRED software. Resulting epitopes were subsequently screened for predicted antigenicity using the VaxiJen resource. BLAST was used to interrogate potential homology between the selected *P. yoelii* epitopes and the *P. falciparum* and *P. vivax* MAEBL antigens).

**Additional file 4.** Alignment of the M2 MAEBL domain sequences of *P. vivax* isolates. (M2 MAEBL amino acid sequences of Brazilian isolates harvested from Manaus (PvBM_1524, 1530, 1209), Mâncio Lima (PvBML-6, 5, 12), Acrelândia (PvBA_32A, 20A, 02A) and Thailand isolates (PvT_VKBT-100, VKBT-101, VKTS-39) compared to P. vivax Sal-1 strain. Sequences of Brazilian isolates were deposited in GenBank with accession numbers: KX061004 to KX061012).
