# Supplementary material for: In silico epitope mapping and experimental evaluation of the Merozoite Adhesive Erythrocytic Binding Protein (MAEBL) as a malaria vaccine candidate
Source: Malar J. 2018 Jan 10;17:20. doi: 10.1186/s12936-017-2144-x (PMC5761135; doi:10.1186/s12936-017-2144-x)
Supplement: Supplementary file 1 — Additional file 1. MAEBL Oligonucleotide position and sequences. (# The nucleotide positions are based on Plasmodium vivax strain Sal-1 MAEBL sequence (PVX_092975) available at PlasmoDB). [file 12936_2017_2144_MOESM1_ESM.docx]

**Additional file 1. MAEBL Oligonucleotide position and sequences**

# The nucleotide positions are based on Plasmodium vivax strain Sal-1 MAEBL sequence (PVX_092975) available at PlasmoDB

| **Fragment** | **Position^#^ (nt)** | **Oligonucleotide** | **Sequence (5’ to 3’)** |
| --- | --- | --- | --- |
| 1 | 1.778 - 2.350 | PvM2-F1 | CCATGCAAAAGAGTGACGAG |
|  |  | PvM2-R1 | GTTCGATACTGCGTCCGTTT |
| 2 | 2.011 - 3.264 | PvM2-F2 | AGATCTAATCCTCAAGCTAAATTTATG |
|  |  | PvM2-R2 | ATTTGTTGTGAGAGATGTGAA |
| 3 | 2.819 - 3.457 | PvM2-F3 | CCGATAACTGCAGAAAGGAA |
|  |  | PvM2-R3 | CCTCTGTTGCACTACCGTCA |
