# Supplementary material for: In silico epitope mapping and experimental evaluation of the Merozoite Adhesive Erythrocytic Binding Protein (MAEBL) as a malaria vaccine candidate
Source: Malar J. 2018 Jan 10;17:20. doi: 10.1186/s12936-017-2144-x (PMC5761135; doi:10.1186/s12936-017-2144-x)
Supplement: Supplementary file 2 — Additional file 2. Predicted MHC class I and II epitopes within the Plasmodium yoelii MAEBL antigen generated from a consensus between different epitope prediction programs. [file 12936_2017_2144_MOESM2_ESM.docx]

**Additional file 2. Predicted MHC class I and II epitopes within the *Plasmodium yoelii* MAEBL antigen generated from a consensus between different epitope prediction programs.**

| Program and Score | | | | | | | | | |
| --- | --- | --- | --- | --- | --- | --- | --- | --- | --- |
| Allele | Position, domain | Epitope sequence | Rankpep | IEDB | NetMHCpan | Bimas | MAPPP | PropredI | NetMHCII |
| H-2-Kb | 420, M1 | QNYYSFTNL | 18.496 | 0.2 | 0.05 | 60.000 | 60 | 4.0943 | ______ |
| H-2-Db | 1005, Inter M2/Repeat | NQNINLVKL | 19.868 | 0.9 | 0.385 | 264.000 | 264 | 5.5759 | ______ |
| I_Ab | 509, Inter M1/M2 | YNYPITPIS | 13.441 | 1.25 | - | - | - | - | 0.20 |
|  | 1354, Inter Repeat/C-cys | FAGAGIIMS | 13.992 | 2.26 | - | - | - | - | 0.40 |
