# Supplementary material for: In silico epitope mapping and experimental evaluation of the Merozoite Adhesive Erythrocytic Binding Protein (MAEBL) as a malaria vaccine candidate
Source: Malar J. 2018 Jan 10;17:20. doi: 10.1186/s12936-017-2144-x (PMC5761135; doi:10.1186/s12936-017-2144-x)
Supplement: Supplementary file 3 — Additional file 3. Predicted Plasmodium yoelii B-cell epitopes within the putative MAEBL antigen. (Epitopes were generated using BCPRED software. Resulting epitopes were subsequently screened for predicted antigenicity using the VaxiJen resource. BLAST was used to interrogate potential homology between the selected P. yoelii epitopes and the P. falciparum and P. vivax MAEBL antigens). [file 12936_2017_2144_MOESM3_ESM.docx]

**Additional file 3. Predicted *Plasmodium yoelii* B-cell epitopes within the putative MAEBL antigen.**

(Epitopes were generated using BCPRED software. Resulting epitopes were subsequently screened for predicted antigenicity using the VaxiJen resource. BLAST was used to interrogate potential homology between the selected *P. yoelii* epitopes and the *P. falciparum* and *P. vivax* MAEBL antigens)

| Position | Homology | Sequence and homology with *P. falciparum* and *P. vivax* (when existing) | BCPRED score | VaxiJen score | Domain within protein |
| --- | --- | --- | --- | --- | --- |
| 37 | *P. falciparum* | ILNNHVNIKWTNSGSLGQGN  \| \|\|\|\|\|\|\|+\|\|\| \|+\|  INKNHVNIKWSNSGIHGKGK | 0.881 | 1.0215 | M1 |
|  | *P. vivax* | ILNNHVNIKWTNSGSLGQGN  \| \|\|\|+\| \|+ +\| \|\|+\|+  IAKNHVDISWSTNGVLGKGD |  |  |  |
| 205 | *P. falciparum* | GSNTEYPLHIYNPIENYRTQ  \|+\| \| \|\|\| \| \|+\| \|+  GANIEEPLHKYKNDEHYVTK | 0.806 | 0.5349 | M1 |
|  | *P. vivax* | GSNTEYPLHIYNPIENYRTQ  \|\|\| \|\|\|\| \|\|\| \|++ \|+  GSNMNYPLHDYNPSESHVTR |  |  |  |
| 233 | *P. falciparum* | ETLEDCSIYSHCIGPCFDRD  + \| \|\|\|\|\|\|\|\|\|\|\|\| +\|  KNLFDCSIYSHCIGPCLYKD | 0.798 | 0.9681 | M1 |
|  | *P. vivax* | ETLEDCSIYSHCIGPCFDRD  + \| \|\|\|\|\|\|\|\|+\|\|\|\|++\|  KNLSDCSIYSHCMGPCFNKD |  |  |  |
| 258 | *P. falciparum* | FRDLPVAFNHKTKECIIIGT  \| +\|\|+ \|\|\|+\|\|\|\|+\|\|\|\|  FLNLPILFNHQTKECVIIGT | 0.827 | 0.9283 | M1 |
|  | *P. vivax* | FRDLPVAFNHKTKECIIIGT  \|\| \|\|\| \|\|\|\|\|\|\|\|+\|+\|\|  FRSLPVVFNHKTKECVILGT |  |  |  |
| 279 |  | EEKKTTNCNSDNSRNNGRCF | 0.997 | 1.1627 | M1 |
| 370 |  | NYANEDPEEKRNNYLWGVWV | 0.986 | 1.4229 | M1 |
| 489 |  | ENSKLQTNKGNETKKTKYGL | 0.978 | 1.2199 | Inter M1/M2 |
| 576 |  | NRNNNYNQPKNKPNPQAEYM | 1 | 0.5579 | Inter M1/M2 |
| 603 | *P. falciparum* | NHIYIDWKQDGKYGSGKLKY  \|\|\|+\|+\|+++\|+\|\|+ + \|\|  NHIFIEWQKEGEYGNDEFKY | 0.987 | 1.1627 | M2 |
|  | *P. vivax* | NHIYIDWKQDGKYGSGKLKY  \|\|\|+\|\|\|\|++\|\| \| \| \|\|  NHIFIDWKKEGKLGEGNFKY |  |  |  |
| 651 | *P. falciparum* | PGRAQGSCPNYGKSIVVKTP  \|\|\| \|\|\|\|\|\|\|\|+\|+\|+-  EGRAHGSCPNYGKAIIVQNL | 0.998 | 0.7809 | M2 |
|  | *P. vivax* | PGRAQGSCPNYGKSIVVKTP  \|\|\|\|\|\|\|\|\|\|\|\|\|+\|+\|+\|-  PGRAQGSCPNYGKAIIVETL |  |  |  |
| 712 |  | HHGDLSVCPKSWDEENLYKK | 0.756 | 0.6344 | M2 |
| 812 | *P. falciparum* | YCLGPCLENAYNNKCFRSLP  \|\|\|\|\|\|\|\|\|++ \|\|\|\|\|+\|\|  YCLGPCLENSFGNKCFRNLP | 0.792 | 0.5146 | M2 |
|  | *P. vivax* | YCLGPCLENAYNNKCFRSLP  \|\|\|\|\|\|+\|\| \| \|\|\|\|\|+\|\|  YCLGPCIENTYKNKCFRNLP |  |  |  |
| 848 | *P. falciparum* | EQERNNNCRTRRSDTDKPNC  \|\|\|\| \|+\|\| + + \|\|\|\|  EQERTNSCRRTKEEKKKPNC | 0.998 | 0.8236 | M2 |
|  | *P. vivax* | EQERNNNCRTRRSDTDKPNC  \|\|\|\| +\|\|\| ++\| \|\|\|\|  EQERTDNCRKEKTDLSKPNC |  |  |  |
| 883 | *P. falciparum* | TSFIRPDYEEKCPPRYPLKF  +\|\|\|\|\|\|\|\| \|\|\|\|\|\|\|\|\|-  SSFIRPDYETKCPPRYPLKS | 0.944 | 0.7185 | M2 |
|  | *P. vivax* | TSFIRPDYEEKCPPRYPLKF  \|\|\|\|\|\|\|\|\|\|\|\|\|\|\|\|\|+--  TSFIRPDYEEKCPPRFPLNS |  |  |  |
| 956 | *P. falciparum* | VWVASESVNSSNLYNAKGEC  +\| \| \|\|\| +\|+ \| \|+\|  IWAADHSVNENNIEIANGKC | 0.984 | 0.5581 | M2 |
|  | *P. vivax* | VWVASESVNSSNLYNAKGEC  \|\|+\|+\|\|\|\| \|+++ \|\|\|  VWIANESVNKDNMFSVNGEC |  |  |  |
| 993 | *P. falciparum* | SFTSLTTNDIDFNQNINLVK  \|\|\|+\|\| \| +\|\|\|\|++\|+ K  SFTALTANTVDFNQSVNIRK | 0.762 | 0.6195 | M2 |
|  | *P. vivax* | SFTSLTTNDIDFNQNINLK  \|\|\|\|\|\|\|\|\|\|\|\| \|\|+\|+-  SFTSLTTNDIDFYQNLNIE |  |  |  |
| 1058 |  | INSYTPNRRGENFAKESDST | 0.93 | 0.8171 | INTER M2/REPEAT |
| 1081 |  | DESKMDEVIRKREEAAKNAE | 0.802 | 0.6225 | INTER M2/REPEAT |
| 1114 |  | AKKAEEERKKAEAVKKAEEE | 0.996 | 1.0350 | REPEAT |
| 1140 |  | EKKAEEERKRIEAEKKAEEE | 1 | 1.1544 | Repeat |
| 1179 |  | AKKAEEERKRIEEAKKAEEE | 1 | 1.0577 | Repeat |
| 1206 |  | KKAEEERKKAEAVKKEEEVI | 1 | 1.0901 | Repeat |
| 1232 |  | SETKISNNYETRNIDDNSFK | 0.896 | 0.7300 | INTER REPEAT/TM |
| 1254 |  | DEEEYKSRNIDNTRNKIISM | 0.993 | 0.7592 | INTER REPEAT/TM |
| 1289 | *P. falciparum* | DYMKDKISSGNCSNDERKQL  \|\|\|\|\| \|\|\|\| \|\|\|+\|\|\| \|  DYMKDNISSGTCSNEERKSL | 0.97 | 1.4943 | INTER REPEAT/TM |
|  | *P. vivax* | DYMKDKISSGNCSNDERKQL  \|\|\| \|\| \|\|\|\|+ \|\|\| \|  DYMMKNISFGNCSDGERKGL |  |  |  |
