# Supplementary figures and images for: In silico epitope mapping and experimental evaluation of the Merozoite Adhesive Erythrocytic Binding Protein (MAEBL) as a malaria vaccine candidate
Source: Malar J. 2018 Jan 10;17:20. doi: 10.1186/s12936-017-2144-x (PMC5761135; doi:10.1186/s12936-017-2144-x)

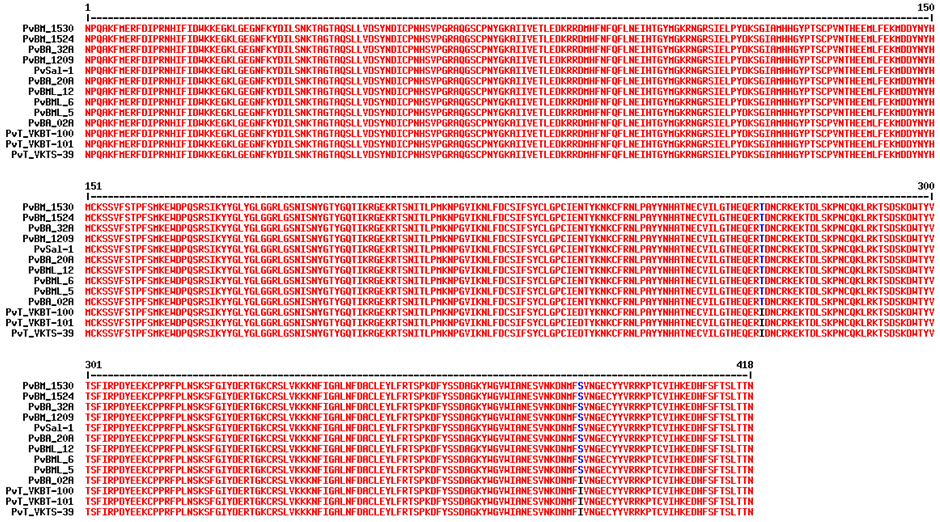

Supplement: Supplementary file 4 — Additional file 4. Alignment of the M2 MAEBL domain sequences of P. vivax isolates. (M2 MAEBL amino acid sequences of Brazilian isolates harvested from Manaus (PvBM_1524, 1530, 1209), Mâncio Lima (PvBML-6, 5, 12), Acrelândia (PvBA_32A, 20A, 02A) and Thailand isolates (PvT_VKBT-100, VKBT-101, VKTS-39) compared to P. vivax Sal-1 strain. Sequences of Brazilian isolates were deposited in GenBank with accession numbers: KX061004 to KX061012). [file 12936_2017_2144_MOESM4_ESM.tif]
